# KiT: a MATLAB package for kinetochore tracking

**DOI:** 10.1093/bioinformatics/btw087

**Published:** 2016-02-15

**Authors:** Jonathan W. Armond, Elina Vladimirou, Andrew D. McAinsh, Nigel J. Burroughs

**Affiliations:** ^1^Division of Biomedical Cell Biology, Mechanochemical Cell Biology Building, Warwick Medical School; ^2^Warwick Systems Biology Centre and Mathematics Institute, University of Warwick, Coventry, CV4 7AL, UK

## Abstract

**Summary:** During mitosis, chromosomes are attached to the mitotic spindle via large protein complexes called kinetochores. The motion of kinetochores throughout mitosis is intricate and automated quantitative tracking of their motion has already revealed many surprising facets of their behaviour. Here, we present ‘KiT’ (Kinetochore Tracking)—an easy-to-use, open-source software package for tracking kinetochores from live-cell fluorescent movies. KiT supports 2D, 3D and multi-colour movies, quantification of fluorescence, integrated deconvolution, parallel execution and multiple algorithms for particle localization.

**Availability and implementation:** KiT is free, open-source software implemented in MATLAB and runs on all MATLAB supported platforms. KiT can be downloaded as a package from http://www.mechanochemistry.org/mcainsh/software.php. The source repository is available at https://bitbucket.org/jarmond/kit and under continuing development.

**Supplementary information:** Supplementary data are available at *Bioinformatics* online.

**Contact:** jonathan.armond@warwick.ac.uk

## 1 Introduction

**Fig. 1.**
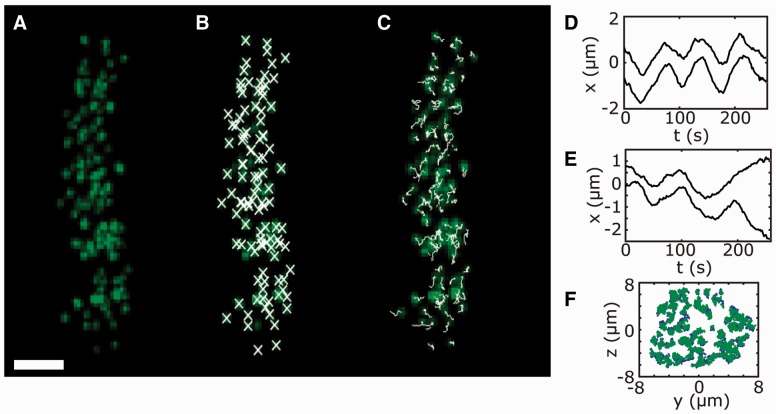
(**A**) Still Z max-projection from metaphase cell movie with kinetochores marked by EGFP-CENP-A (Enhanced Green Fluorescent Protein-CENtromere Protein A), with **(B)** particles marked by crosses or **(C)** tracks formed up to this frame marked by lines. Scale bar 3 μm. **(D)** An example metaphase kinetochore track from cell in (A). **(E)** An example metaphase to anaphase transition track from a different cell. **(F)** Metaphase plate view of kinetochore trajectories from cell in (A)

The process of mitosis involves the attachment of chromosomes to a protein scaffold, called the mitotic spindle, via large protein complexes called kinetochores (McIntosh *et al.*, 2012). Throughout mitosis, the kinetochores, and hence the chromosomes, execute a highly complex stochastic motion culminating in the segregation of the genetic material to the two daughter cells. Automated quantitative tracking of fluorescently labelled kinetochores is revealing surprising details of their behaviour and makes the analysis of large datasets consisting of hundreds or thousands of cells feasible (Armond *et al.*, 2015a,b; Burroughs *et al.*, 2015; Jaqaman *et al.*, 2010; Kitajima *et al.*, 2011; Vladimirou *et al.*, 2013). We have developed a MATLAB package—Kinetochore Tracking (KiT)—for tracking the motion of kinetochores, facilitating the quantitative analysis of chromosome motion Fig. 1A–C. Although KiT is primarily developed with tracking of kinetochores in mind, it is also useful as a tool for tracking other fluorescently-marked particles in cells, e.g. centrosomes, motors etc., provided they have an approximately Gaussian shape and are not extended objects. For example, the centrosomes can also be tracked when fluorescently marked (e.g. Burroughs *et al.*, 2015).

## 2 Features

KiT evolved from earlier software for particle tracking (u-Track; Jaqaman *et al.*, 2008), kinetochore tracking (MaKi; Jaqaman *et al.*, 2010) and kinetochore track analysis (CupL; Vladimirou *et al.*, 2013) and incorporates numerous enhancements and major new features. Movies are loaded through the Bioformats package (Linkert *et al.*, 2010), which is automatically downloaded, enabling compatibility with a vast range of microscopy data formats.

### 2.1 Graphical User Interface

KiT includes a user-friendly GUI (Graphical User Interface) for selecting ROIs (Regions Of Interest; to select cells and exclude spurious background fluorescence), parameter configuration and execution of KiT. Tracking may be executed from within the GUI or later. The GUI allows selection of particle detection algorithms per channel and modification of the most commonly used options. KiT also includes a GUI for post-tracking processing, enabling basic diagnostics and track quantification, such as kinetochore speeds and autocorrelation. The GUI collates data from each tracked ROI and saves a .mat file for later user-specific processing.

### 2.2 Multi-channel tracking

Often the correlative dynamics of multiple proteins is of interest. To facilitate this, KiT allows independent tracking of objects in different channels. To allow correlation between the dynamics of multiple channel data, a coordinate system is derived from a single selected channel and the other channels are transformed into this system. An option is also provided to supply an experimentally determined point-spread function (PSF), which is then used to perform deconvolution via the MATLAB function deconvlucy.

### 2.3 Fluorescence quantification

In addition to multi-channel tracking, fluorescent intensity is measured within a customizable mask over the tracked object in all channels. For example, we have quantified EB3-EGFP levels at kinetochores which were tracked by mCherry-CENP-A (Armond *et al.*, 2015b).

### 2.4 Modular detection algorithms

The signal-to-noise ratio of live-cell imaging data can vary widely. To accommodate this, we introduced a modular system for choosing particle detection algorithms. In the primary stage, particle locations are initially detected using: (a) unimodal histogram thresholding (Rosin, 2001), (b) multiscale wavelet product thresholding (Olivo-Marin, 2002) or (c) point-cloud similarity adaptive thresholding. Algorithm (c) is a novel method for setting a global threshold based on maintaining stability in the number and location of particles (see Supplementary Materials). Algorithm (a) is the fastest but least robust to background fluorescence (e.g. bimodal histograms), while (b) is slower and requires more fine-tuning but is capable of excluding more false positives in noisy images. Algorithm (c) offers a balance between speed and sensitivity (see Supplementary Figure S1).

After initial detection, an optional stage of location refinement is available using either: (d) calculation of the centroid of the particle (Jaqaman *et al.*, 2010), or (e) fitting a mixture model of 3D Gaussian functions to the spot locations (Thomann *et al.*, 2002) within regions of potentially overlapping PSFs, in order to allow the discrimination of sub-resolution particles. Algorithm (e) is significantly slower than (d), thus we allow the independent selection of initial particle detection and refinement for those applications where subpixel-resolution is not required.

### 2.5 Coordinate systems

Cells and the mitotic spindle itself are prone to translation and rotation. We have found that different reference frames for defining a coordinate system are useful in different circumstances. KiT provides three options: (i) metaphase plate, where the kinetochores are aligned such that variation along two principal axes is much larger than along a third (Jaqaman *et al.*, 2010), (ii) image moments, similar to (i) except we use the eigenvectors of the image covariance matrix instead of particle locations, which is useful for 2D movies (Armond *et al.*, 2015b), (iii) translation correction only.

### 2.6 Parallel execution

On a 2.9 GHz MacBook Pro, tracking of a 150 frame movie takes ∼5 min per ROI using refinement algorithm (d), and around 30 min using (e). The total analysis time of large datasets may be reduced by invoking the parallel execution option, which uses the batch processing facility of the MATLAB Parallel Computing Toolbox to schedule each ROI to run in parallel on a multi-core machine.

## 3 Usage

No installation is necessary; the source directory may optionally be added to the MATLAB path. The process of tracking in a single ROI is termed a job, and the tracking of a whole set of ROIs is termed a jobset. To setup a jobset the following command is used: jobset = kitGUI. This results in the GUI display where the user can select the movies to be analyzed, select ROIs from each movie, specify the tracking requirements and customize various options. After choosing a jobset name and completing the tracking setup, the jobset description file is saved to disk. The tracking is started from within the GUI or with the command: kitRunJobs(jobset). For each ROI, a file is generated containing the kinetochore tracks and other data. Simple analysis can be performed on the data (see examples in Fig. 1D–F), e.g. plotting kinetochore tracks or computing the autocorrelation, with the command: kitAnalysis(jobset). This opens a GUI with buttons for each analysis plot. An option is provided to save a .mat file containing collated data from across all ROIs for external processing.

## 4 Conclusion

We have developed KiT, a software tool which enables user-friendly and efficient particle tracking and basic analysis of kinetochores and other objects. We hope that it will lower the entry barrier for quantitative tracking analysis of live-cell imaging data by cell biology laboratories.

## Supplementary Material

Supplementary Data
