# Supplementary material for: KiT: a MATLAB package for kinetochore tracking
Source: Bioinformatics. 2016 Feb 15;32(12):1917–9. doi: 10.1093/bioinformatics/btw087 (PMC4908324; doi:10.1093/bioinformatics/btw087)
Supplement: Supplementary Data [file supp_32_12_1917__index.html]

KiT: a MATLAB package for kinetochore tracking — KiT: a MATLAB package for kinetochore tracking — Supplementary Data 

# KiT: a MATLAB package for kinetochore tracking

## Supplementary Data

files

- Supplementary Data - pdf file
