# Supplementary material for: KiT: a MATLAB package for kinetochore tracking
Source: Bioinformatics. 2016 Feb 15;32(12):1917–9. doi: 10.1093/bioinformatics/btw087 (PMC4908324; doi:10.1093/bioinformatics/btw087)
Supplement: Supplementary Data [file supp_btw087_kit_supp_rev1_v1.pdf]

Supplementary information for KiT: A MATLAB package for kinetochore tracking

Jonathan W. Armond, Elina Vladimirou, Andrew D. McAinsh and Nigel J. Burroughs

1 Point cloud similarity adaptive threshold algorithm

The point cloud similarity adaptive threshold algorithm is a particle detection algorithm that incorporates the temporal structure of the data to improve discrimination between true and false positives. It is predicated on the assumption that true particles do not make very large displacements between frames and that false positives, due to noise, have uncorrelated positions between frames. Thus, the algorithm aims to set a global intensity threshold (Sahoo *et al.*, 1988) which minimizes the difference between the cloud of particles in one frame and the next (Torre-Ferrero *et al.*, 2012), while simultaneously maximizing the number of particles accepted.

Firstly, the background levels present in the movie are estimated using a 3D Gaussian filter with a large variance ($\sigma = 16$, in pixel units) and filter window (63x63xZ, where Z is the number of Z-planes) and subtracted. Local maxima r_i^t are then located within each frame t and the intensity I_i^t for each maximum i recorded. Photobleaching is estimated by fitting an exponential function to the mean intensity in each image frame and used to correct the intensity of the local maxima. The set of detected particles for frame t is defined as $P^t = \{r_i^t : I_i^t \geq \tau\}$, where I_i^t are taken here as the corrected maxima intensities and τ is an intensity threshold. The threshold is determined by globally minimizing an objective function $f(\tau)$ using a pattern search algorithm (using the `patternsearch` function from the MATLAB Global Optimization Toolbox). We defined the objective as

$$f(\tau) = \frac{1}{N-1} \sum_{j=1}^{N-1} M(P^j, P^{j+1}) + \lambda \left[\max\left(1, \frac{1}{N} \sum_{j=1}^{N} |P^j| \right) \right]^{-K} \tag{1}$$

where N is the number of frames and $|\cdot|$ indicates the size of a set. The function M accounts for the overall change in positions between two sets of maxima (i.e., point clouds) A and B and is defined as]

$$M(A, B) = \frac{1}{|A| + |B|} \left[\sum_{a \in A} \min d(a, B) + \sum_{b \in B} \min d(A, b) \right] \tag{2}$$

where d is the Euclidean distance function. The second term of $f(\tau)$ is larger for fewer particles and therefore penalises overly stringent thresholds. The parameters λ and K are for regularization and penalty strength, respectively. We found $\lambda = 10$ and $K = 1$ to be effective.

2 Simulated particle grids

To demonstrate the performance of the three particle detection algorithms incorporated in KiT, we generated synthetic images in MATLAB (Figure 1). Beginning with a completely dark background, we set pixels on regularly-spaced lattice to a non-zero intensity such that pixels at the right of the lattice were brighter than those on the left, in a linear fashion. To be precise, pixels in lattice column y were given intensity $I(y) = y/M$, where M is the number of columns, and the maximum intensity is 1. We then convolved the image with a Gaussian filter to simulate the effect of a microscope point spread function (PSF).

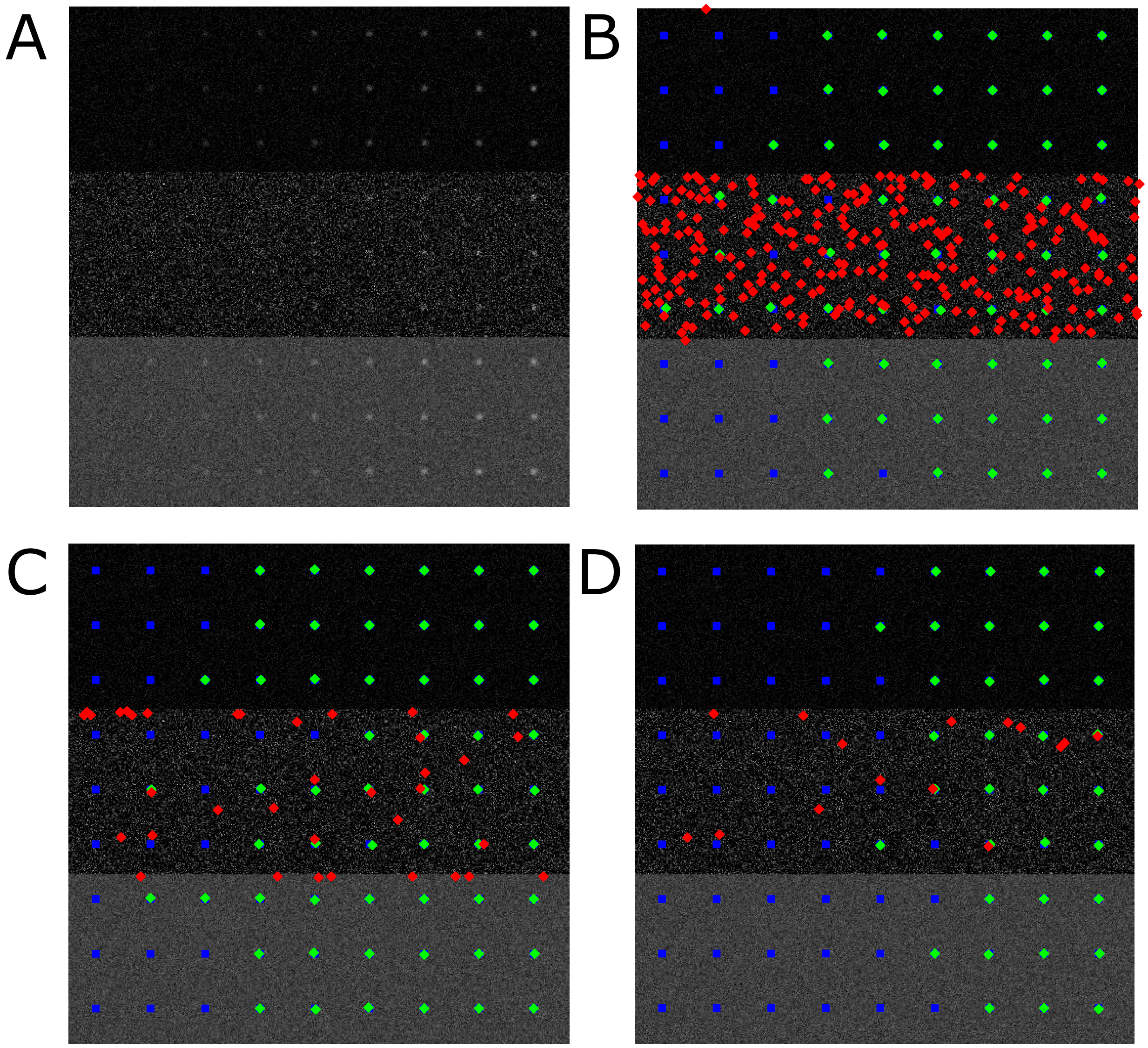

Figure 1: **Comparison of particle detection algorithms**. (A) Image is a simulated particle grid generated by adding bright points with a decaying intensity from right to left and then convolving with a Gaussian filter. Three Gaussian noise bands were then added: zero mean low variance (top), zero mean high variance (middle), nonzero mean low variance (top). Three algorithms were applied to the image in (A) and are: unimodal histogram thresholding (B), multiscale wavelet product thresholding (C), point-cloud similarity adaptive thresholding (D). (N.b., since the point-cloud similarity algorithm minimizes the difference in the particle locations between successive frames, it does not operate on single frames, thus we generated another similar grid to use in place of a second frame). Blue dots indicate true particle positions, green/red are true/false positive detection, respectively.

We then split this base image, which consists of rows of identical simulated particles, into three equal horizontal bands and added three different noise distributions, η_i:

- **Top:** Zero mean, low variance Gaussian noise: $\eta_1 \sim N(0, 0.005)$
- **Middle:** Zero mean, high variance Gaussian noise: $\eta_2 \sim N(0, 0.05)$
- **Bottom:** Non-zero mean, low variance Gaussian noise: $\eta_3 \sim N(0.25, 0.005)$

In the bottom band, to compensate for the additive increase in brightness, we first reduced the band to 75% intensity. Finally, we added Poisson noise to all the bands, $\eta_P(x, y) \sim Poi(\lambda)$, where the mean λ is proportional to the pixel intensity, i.e. $\lambda \propto I(x, y)$.

References

Sahoo, P. K., Soltani, S., Wong, A. K. C., and Chbn, Y. C. (1988). Survey of Thresholding Techniques. *Comput. Vision, Graph. Image Process.*, **41**, 233–260.

Torre-Ferrero, C., Llata, J. R., Alonso, L., Robla, S., and Sarabia, E. G. (2012). 3D point cloud registration based on a purpose-designed similarity measure. *EURASIP J. Adv. Signal Process.*, **2012**, 57.
